# Role of trypsin and protease-activated receptor-2 in ovarian cancer

**DOI:** 10.1371/journal.pone.0232253

**Published:** 2020-05-04

**Authors:** Kyu Kwang Kim, Rachael Turner, Negar Khazan, Arif Kodza, Aaron Jones, Rakesh K. Singh, Richard G. Moore

**Affiliations:** The Wilmot Cancer Institute at the University of Rochester Medical Center, Rochester, NY, United States of America; The University of Hong Kong, HONG KONG

## Abstract

Proteases have been implicated in the tumorigenesis and aggressiveness of a variety of cancer types. In fact, proteases have proven to be very clinically useful as tumor biomarkers in the blood of patients. Proteases are typically involved in complex systems of substrates, activators, and inhibitors, thus making our ability to establish their exact function in cancer more difficult. Trypsin, perhaps the most famous of proteases, has been shown to play a role in cancer progression, but its functional role in ovarian cancer has not been much studied. PAR2, a transmembrane receptor that is known to be activated by trypsin, has been reported to be associated with ovarian cancer. Here, we found that stimulation of ovarian cancer cell lines with trypsin or PAR2 activating peptide markedly increased MAPK signaling and cell proliferation. Additionally, HE4, a WAP-family glycoprotein and ovarian cancer biomarker, was found to inhibit trypsin degradation, thereby retaining its activity. Patient data seemed to support this phenomenon, as the serum of ovarian cancer patients with high HE4 expression, revealed significantly elevated trypsin levels. These data support the hypothesis that trypsin plays a tumorigenic role in ovarian cancer, which can be mediated by its receptor PAR2, and potentiated by HE4.

## Introduction

Proteases are important to a number of complex processes in the human body and to date, over 500 distinct proteases have been identified. Proteases are critical to ovulation, enteral digestion, inflammation/immunity, wound healing, angiogenesis and coagulation. They typically operate in complicated systems of substrates, inhibitors, receptors, adaptors, and cofactors [1]. While proteases are essential in the maintenance of healthy cells and organ function, they have also been shown to be overexpressed in multiple cancer subtypes [2]. Cancer related proteases and protease inhibitors can be found in the serum of patients, and have been utilized as diagnostic and prognostic biomarkers; examples include prostate-specific antigen, plasminogen activator and cathepsins [3]. Both extracellular and intracellular proteases are known to play a role in cancer cells [4]. While their exact contribution to tumorigenesis has yet to be fully elucidated, several proteases have been shown to be crucial for tumor angiogenesis, invasion, and metastasis [5].

Trypsin, a serine protease, is one of the most heavily studied proteases best known for its role in enteric digestion. Trypsinogen, the proform of trypsin, is synthesized by the acinar cells of the pancreas, secreted into the duodenum, and activated by enterokinase. Extra-pancreatic expression of trypsin has been reported in several cancer types [6–9], and its tumorigenic role has been described in both *in vivo* and *in vitro* models [7, 10, 11]. In ovarian cancer, the expression of trypsinogen is associated with tumor aggressiveness [12, 13]. Trypsin (ogen) or trypsin-like activity is found in ovarian cancer cyst fluid, serum and ascites [12, 14–16]. Trypsin is known to degrade a wide variety of extracellular matrix (ECM) components [17], and to induce activation cascades of other proteases, most notably, matrix metalloproteinases (MMP) [14] and urokinase-plasminogen activators [18], which promote ovarian tumor invasion [19, 20].

Protease activated receptors (PARs) are a family of the seven transmembrane G protein-coupled receptors that are activated by serine proteases. PARs consist of four isoforms [21–23]: PAR2 is activated by trypsin and PAR1,3 and 4 are all activated by thrombin [24]. Unlike canonical receptor activation via ligand-receptor interaction, PAR2 is activated by a proteolytic mechanism in which the PAR2 agonist (i.e. trypsin) binds to and cleaves the amino-terminus of the receptor. This receptor cleavage generates a tethered ligand sequence, such as SLIGKV, that binds to and activates the core receptor [21, 22, 25, 26]. PAR2 expression has been observed in several cancer types, including ovarian cancer, where its expression is associated with tumor aggressiveness [27, 28]. In gynecologic cancers specifically, PAR2 has been found to promote cancer cell proliferation, invasion, migration and metastasis [10, 28]. The exact role of trypsin-PAR2 signaling has not been fully elucidated in ovarian cancer, but PAR2 has been associated with increased IL-8, VEGF, and MMP activity [10, 28]. The study described here was designed to evaluate the tumorigenic potential of trypsin and PAR2 activation in epithelial ovarian cancer (EOC).

## Results

### Expression of PAR2 and trypsin in ovarian cancer

**Fig 1:**
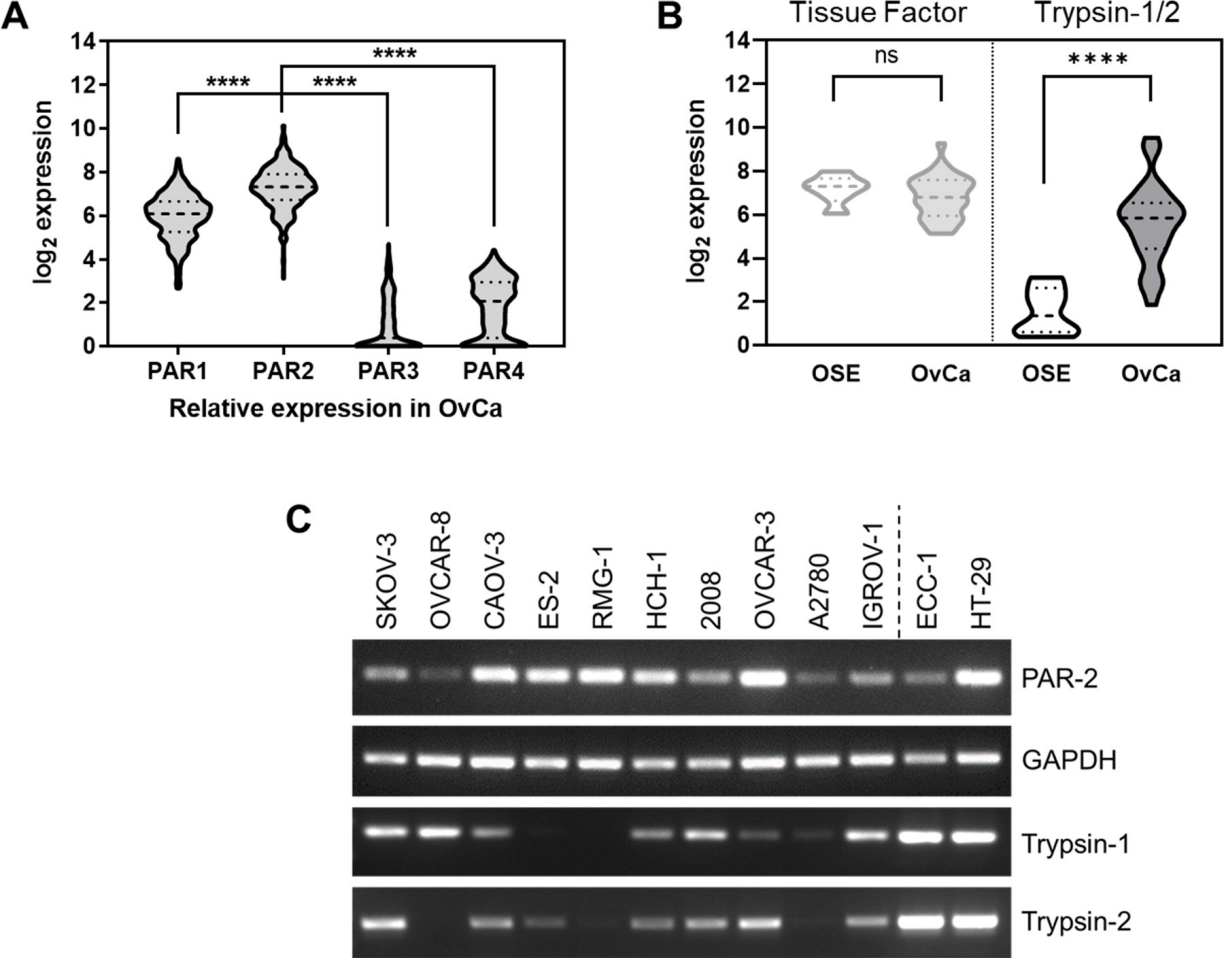
PAR2 and trypsin expression in ovarian cancer. (A) Relative gene expression of PARs in ovarian cancer was analyzed using the TCGA dataset (Tumor Ovarian Serous Cystadenocarcinoma-TCGA-527-MAS5-htu133a; *N* = 509); ****: *p*<0.0001: unpaired t test (PAR2 vs. PAR1) or Welch’s t test (PAR3 vs. PAR2; PAR4 vs. PAR2); Violin plot: median (dashed line); quartiles (dotted line). Similar results were obtained from other ovarian cancer datasets (Tumor Ovarian-Bowtell-285-MAS5.0-u133p2; Mixed Ovarian Cancer (CAFs)-Wong-77-MAS5.0-u133p2; Mixed Ovarian-Birrer-63-MAS5.0-u133p2); See S2 Fig. (B) Tissue distribution of PAR2 activating factors between ovarian surface epithelium and ovarian cancer tissues was analyzed from the dataset, which contains both tissue types; Mixed Ovarian Cancer (CAFs)-Wong-77-MAS5.0-u133p2 (OSE: microdissected ovarian surface epithelium, *N* = 6; OvCa: microdissected ovarian tumor epithelial component, *N* = 32); ns: not significant; ****: *p*<0.0001 (unpaired t test). Similar result was obtained from another ovarian cancer dataset (Mixed Ovarian-Birrer-63-MAS5.0-u133p2; See S2 Fig). (C) Expression of PAR2 and trypsin-1/2 in ovarian cancer cell lines was determined by semiquantitative RT-PCR. Expression of GAPDH served as a loading control; ECC-1 (endometrium) and HT-29 (colon) cancer cells were included as a positive control.

Relative expression of PAR isoforms in ovarian cancer was retrieved from The Cancer Genome Atlas (TCGA) and three other publicly accessible ovarian cancer datasets. Comparatively, PAR2 exceeds the expression levels of all other PARs (Figs 1A and S1), consistent with the previous report [28]. Tissue Factor (TF)-FVIIa is known to induce PAR2 activation in ovarian cancer [28], so relative expression of TF or trypsin-1/2 (encoded by *PRSS1/2)*, which accounts for the majority of trypsin isoforms [29], was compared between ovarian surface epithelium (OSE) and ovarian cancer tissues. Trypsin expression was significantly higher in ovarian cancer tissues than in OSE (Fig 1B), whereas no significant difference was found in TF expression. Similar results were found in a second ovarian cancer dataset (S2 Fig).

### Activation of PAR2 induces cell proliferation in ovarian cancer cell lines

**Fig 2:**
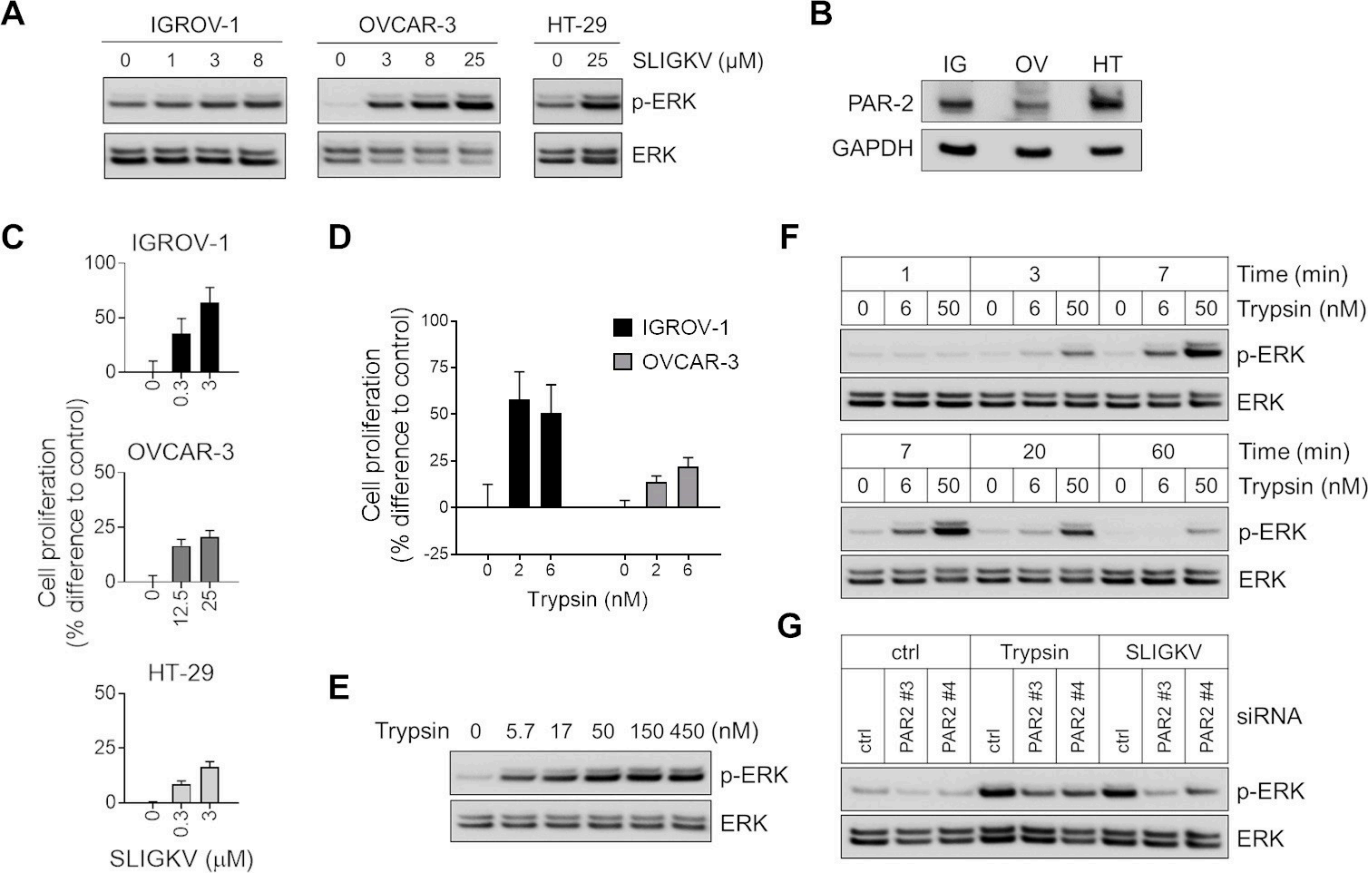
PAR2 agonist and trypsin induce PAR2 signaling in ovarian cancer cell lines. (A) Cancer cells were stimulated with PAR2 activating peptide (SLIGKV-NH_2_) for 7 min. The levels of ERK or phosphorylated ERK were measured by Western blot analysis. (B) Expression of PAR2 in cancer cell lines was determined by Western blot analysis. IG: IGROV-1, OV: OVCAR-3, HT: HT-29. (C) Cancer cells were stimulated with SLIGKV-NH_2_ for 96 h (OVCAR-3 and HT-29) or 120 h (IGROV-1), after which cell proliferation was measured by MTS assay. (D) Ovarian cancer cell lines were stimulated with trypsin at the indicative concentrations for 96 h, after which cell proliferation was measured by MTS assay. (E) OVCAR-3 cells were stimulated with trypsin for 7 min at the indicated concentrations. (F) OVCAR-3 cells were stimulated with the fixed concentrations of trypsin (6 or 50 nM) for the duration as indicated. (G) PAR2 knockdown in OVCAR-3 cells was achieved by transient transfection with siRNAs targeting PAR2 or with non-targeting control siRNA (50 nM). At 48 h post transfection cells were serum starved for 6 h and stimulated with either trypsin (6 nM) or SLIGKV (3 μM) for 7 min. The levels of ERK or phosphorylated ERK were determined by Western blot analysis.

The gene expressions of PAR2 and trypsin-1/2 in ten different ovarian cancer cell lines were determined by reverse-transcription PCR (RT-PCR). We found that a majority of cell lines expressed trypsin(s) and PAR2 (Fig 1C), but RMG-1 lacked expression of both trypsin-1/2. To evaluate the functional role of PAR2 in cells, we tested IGROV-1 and OVCAR-3 ovarian cancer cell lines. Since the biological activity of PAR2 signaling has been determined in HT-29 colon cancer cell line, we included HT-29 cells as a control [24]. We incubated these cells with PAR2 agonist peptide SLIGKV [24] and determined that this exposure results in the phosphorylation of the extracellular signal-regulated kinase ERK (Fig 2A). The PAR2/ERK signaling axis is known to induce cellular proliferation [24]. We found that activation of PAR2 by SLIGKV promotes cell proliferation in IGROV-1 and OVCAR-3 cells (Fig 2C). Likewise, trypsin stimulation increased the proliferation of the same cells (Fig 2D). Western blot analysis revealed that trypsin induces a dose-dependent (Fig 2E) and time-dependent activation of ERK in OVCAR-3 cells (Fig 2F) while a PAR2 knockdown attenuated the activation of ERK by trypsin or SLIGKV (Fig 2G).

### The effects of trypsin degradation on cancer cell proliferation

**Fig 3:**
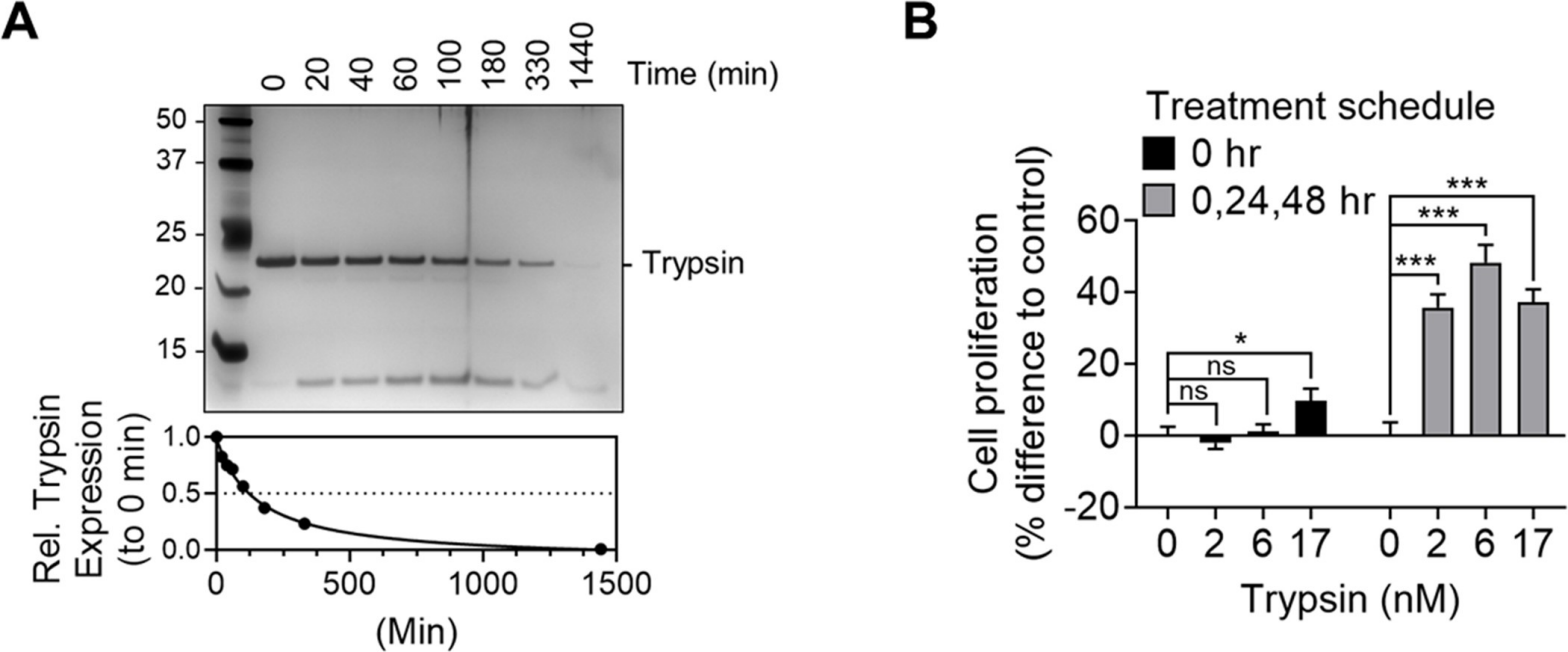
Downregulation of trypsin. **(**A) Trypsin (150 nM) in DMEM culture medium was incubated at 37°C for the indicated durations, after which trypsin expression was analyzed by SDS-PAGE electrophoresis with silver staining; 1^st^ lane: protein size maker (kDa); T_1/2_ (the time for degradation of a half of trypsin) = 122 min. (B) OVCAR-3 cells received one-time stimulation with trypsin and further incubated for 72 h or cells were stimulated with trypsin daily for 72 h, after which cell proliferation was determined by MTS assay. ns: not significant; *: *p*<0.05: ***: *p*<0.001 (two tailed, homoscedastic T-test).

Self-degradation of trypsin is well-studied [30]. To determine its half-life (T_1/2_) under our experimental condition, we incubated trypsin in Dulbecco’s Modified Eagle Medium at 37°C for different durations, after which trypsin expression was analyzed by SDS-PAGE electrophoresis with silver staining. We found that T_1/2_ for trypsin was 122 minutes (Fig 3A). The effects of trypsin degradation on cell proliferation was then assessed. When OVCAR-3 cells were stimulated with trypsin once and left for further incubation for three days, we detected only a marginal increase in their proliferation (Fig 3B, at 17 nM) whereas daily stimulation for three days markedly enhanced the proliferation more than 40 percent (Fig 3B).

### HE4 potentiates trypsin activity

**Fig 4:**
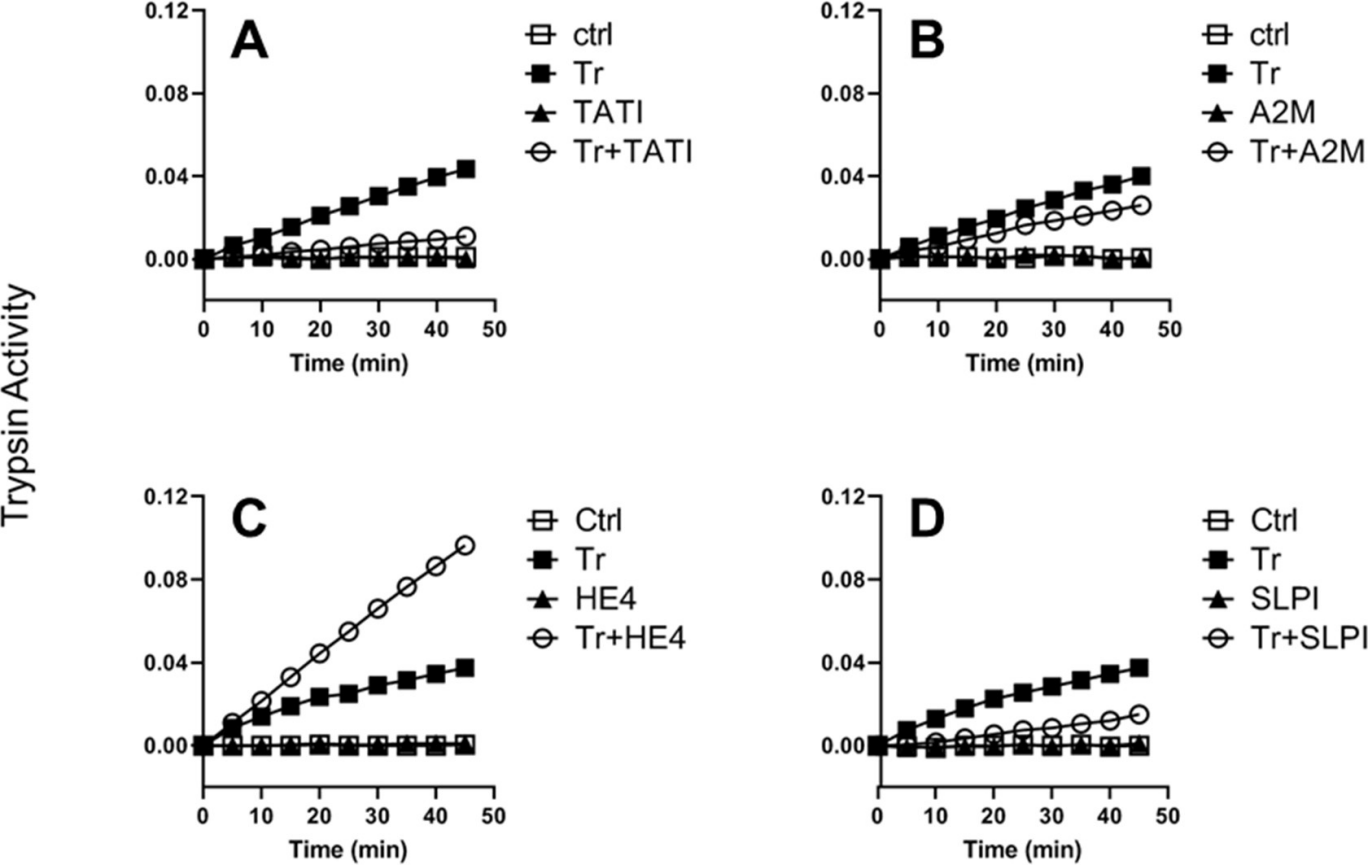
HE4 potentiates trypsin activity. Trypsin activity was measured by the proteolytic cleavage of its substrate (*N*_α_-benzoyl-DL-arginine *p*-nitroanilide). The release of *p*-nitroanilide was colorimetrically detected (λ = 415 nm). Y-axis: value–baseline. Tr: trypsin; TATI: tumor-associated trypsin inhibitor; SLPI: secretory leukocyte protease inhibitor; A2M: α-2-macroglobulin. See Methods for detailed protocols.

Once secreted and activated, trypsin can be neutralized by tissue specific environmental factors. Thus, we conducted a trypsin activity assay in the presence of putative trypsin inhibiting factors implicated in ovarian cancer. Trypsin activity is known to be inhibited by tumor-associated trypsin inhibitor (TATI) [16], which we confirmed in a cell-free trypsin activity assay (Fig 4A). α-2-macroglobulin (A2M) can also rapidly neutralize activated trypsin [16]; when tested in the trypsin assay, a marginal inhibition of trypsin activity by A2M was also seen (Fig 4B). HE4, a WAP-family protein highly upregulated in ovarian cancer [31], is predicted to function as an anti-serine protease [32, 33]. Surprisingly, we observed that HE4 markedly enhances the activity of trypsin (Fig 4C). This finding held true with recombinant HE4 obtained from a different manufacturer (S3 Fig). We then tested another WAP protein with known anti-protease activity; secretory leukocyte protease inhibitor (SLPI) [34]. As expected, SLPI clearly reduced the activity of trypsin (Fig 4D).

### HE4 enhances trypsin integrity

**Fig 5:**
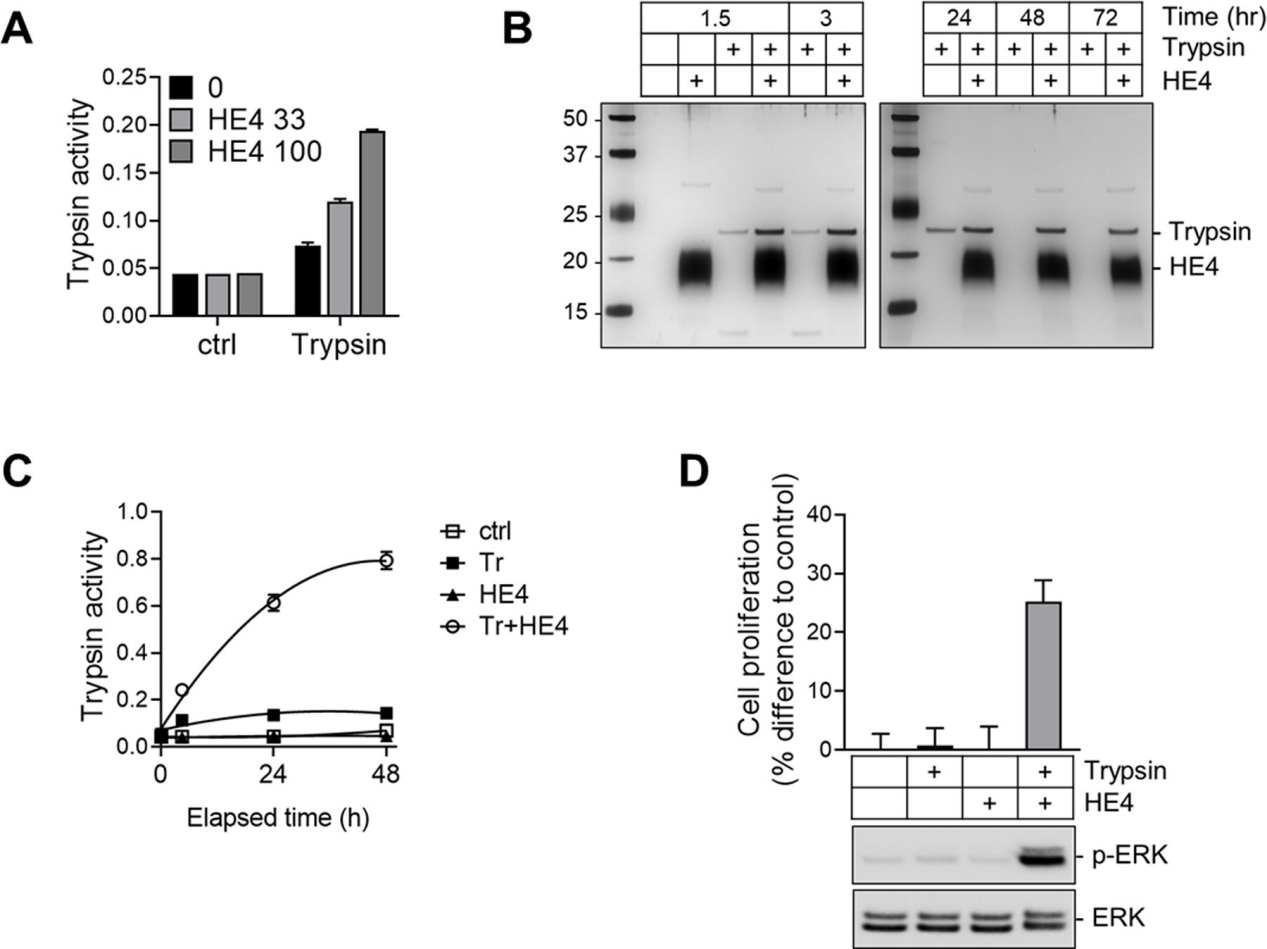
HE4 enhances trypsin integrity. (A) Trypsin (75 nM) was coincubated with HE4 (at 33 or 100 nM). Trypsin activity was measured by the proteolytic cleavage of its substrate (*N*_α_-benzoyl-DL-arginine *p*-nitroanilide). The release of *p*-nitroanilide was colorimetrically detected. (B) Trypsin (150 nM) and/or HE4 (100 nM) was incubated at 37°C in DMEM culture medium for the durations as indicated, after which trypsin or HE4 expression was analyzed by SDS-PAGE electrophoresis with silver staining; 1st lane: protein size maker (kDa). (C) Residual trypsin activity was monitored by the aforementioned procedure in (A). Tr: trypsin (75 nM); HE4 (100 nM). (D) Trypsin and/or HE4 was preincubated at 37°C in DMEM culture medium for 24 h prior to stimulation to OVCAR-3 cells for 72 h (top) or 7 min (bottom). cell proliferation was measured by MTS assay (top). The levels of ERK or phosphorylated ERK were determined by Western blot analysis (bottom). Final treatment concentrations: Trypsin (17 nM); HE4 (30 nM).

**Fig 6:**
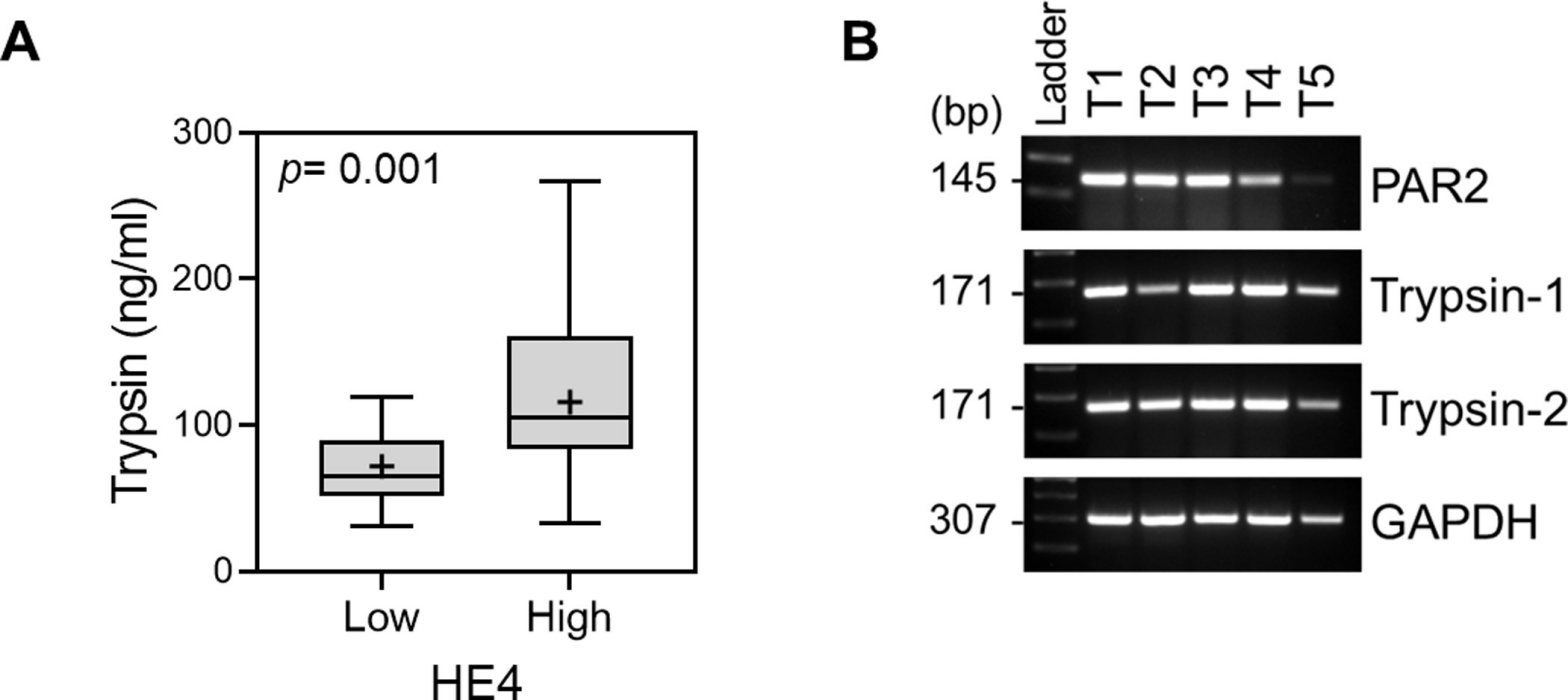
Trypsin levels are elevated in a group with higher HE4. (A) Serums from patients with ovarian cancer were analyzed for trypsin expression by human pan-trypsin specific enzyme-linked immunosorbent assay. See Methods for detailed protocols. Each HE4 group was stratified by pre-determined HE4 concentrations (HE4 cut-off = 100 pM). unpaired, two-tailed Welch’s t test (high vs. low); Sample size: HE4 high (*N* = 28); HE4 low (*N* = 15). (B) Expression of PAR2 and trypsin-1/2 in ovarian cancer patient tissues (T1-T5) was determined by semiquantitative RT-PCR. GAPDH expression served as a loading control.

To understand whether HE4 enhances trypsin activity in a dose-dependent manner, we co-incubated trypsin with different concentrations of HE4. We found that trypsin activity increased with the higher concentration of HE4 (Fig 5A). We then incubated trypsin with or without HE4 for different durations, after which expressions of both proteins were analyzed by SDS-PAGE electrophoresis with silver staining. The presence of HE4 enhanced trypsin integrity at the 1.5-hour co-incubation (Fig 5B), and it lasted for a 72-hour period compared to cultures without HE4. HE4 steadily increased trypsin activity over a 48-hour period, while the activity of trypsin alone plateaued within a few hours (Fig 5C). To this end, we pre-incubated trypsin in culture media with or without HE4 for 24 hours prior to stimulating OVCAR-3 cells. We found that trypsin in the presence of HE4 appeared to retain its activity, thus it was able to promote the proliferation of the cells (Fig 5D Top), and to induce ERK phosphorylation (Fig 5D Bottom). In order to evaluate the possible *in vivo* relevance of our findings, we asked whether PAR2 and trypsin are expressed in tissue samples. As shown in Fig 6B, we detected the expression of PAR2 and trypsin-1/2 using RT-PCR in ovarian cancer patient tissues. Additionally, we analyzed trypsin and HE4 levels in serums from ovarian cancer patients. Our data showed that trypsin levels are elevated in a group of samples with higher HE4 concentrations (Fig 6A).

## Discussion

The tumorigenic role of trypsin has been investigated in several cancer types [7, 10, 11], but to our knowledge this is the first report describing a tumorigenic potential of trypsin in ovarian cancer. The present study shows that the expression of trypsin is higher in ovarian cancer tissues than in OSE tissues (Figs 1B and S2), and that multiple EOC cell lines express trypsin (Fig 1C). Enhanced trypsin expression has been correlated to tumor aggressiveness [13]. In advanced EOC, serum concentration of trypsin-2 complex is an unfavorable prognostic factor, suggesting the adverse role of trypsin in advanced disease [16].

The ERK signaling cascade is known to be associated with cell survival, proliferation and drug resistance in cancer cells [35]. ERK phosphorylation was required for PAR2-mediated cell proliferation [24, 36]. Our study here also found that PAR2 activation induces ERK activation and increases cell proliferation in ovarian cancer cell lines. While it was not investigated here, PAR2 is also known to enhance tumor cell invasive and angiogenic activity, which may increase the aggressiveness of PAR2 presenting tumors. In colon cancer, PAR2 enhanced cancer cell growth through the signaling axis consisting of PAR2, MMPs, TGF-α, EGFR and ERK [24]. It is interesting that all of these pathway constituents are known to be crucial in the progression of ovarian cancer [37, 38].

HE4 is a secretory glycoprotein that is highly upregulated in ovarian and endometrial cancers [31, 39] and, like trypsin, correlates to more advanced and aggressive disease [40]. In a healthy tissue, HE4 likely plays important roles in processes such as sperm maturation [41] and respiratory tract innate immunity [42], but we do not yet have a thorough understanding of its biological function in a malignant tissue. HE4 is encoded by the WAP-type Four-Disulfide Core 2 gene (*WFDC2)* and its highly conserved signature WAP motif is suggestive of anti-protease function [32, 33]. Indeed other WAP proteins, namely SLPI and elafin, are well known for their serine protease inhibition activity [34]. Surprisingly, in direct contrast to the activity of its WAP family members, our study suggested that physiologic levels of HE4 could potentiate the proteolytic activity of trypsin. While it has been shown that extremely high (micromolar) HE4 concentrations can cause moderate trypsin inhibition [43], we argue that our findings are more relevant since they demonstrate the function of HE4 at concentrations akin to what would be found in the ovarian cancer tumor microenvironment. This protease potentiation could be occurring, at least partly, by HE4’s ability to decrease trypsin self-degradation (Fig 5B and 5C) and thereby increase its functional activity (Fig 5D). Interestingly, we also detected elevation of trypsin levels in a group of ovarian cancer patients presenting high levels of serum HE4. However, serum trypsin could be affected by other physiological events, independent of HE4, or by non-malignant tissues. Further study will be needed to confirm our findings by applying a more rigorous model that better reflects a cellular and physiological context.

In summary, our data demonstrated that HE4 augments the activity of trypsin, thereby enhancing PAR2 signaling, and leading to increased ovarian cancer cell proliferation. While the potentiation of trypsin by a predicted protease inhibitor seems counter-intuitive, HE4 was found to inhibit trypsin degradation, increasing its stability and ultimately its enzymatic potential. These findings have implications on future therapeutic design in ovarian cancer and lend credence to the strategies of targeted inhibition of either HE4, trypsin or PAR2.

## Methods

### Cell lines, cell culture and reagents

A2780 cell line was purchased from Sigma-Aldrich. 2008 cell line was kindly provided by Dr. François X. Claret (University of Texas M. D. Anderson Cancer Center). RMG-1 and HCH-1 were a kind gift of Dr. Hiroaki Itamochi (Tottori University, Japan). All other cell lines were purchased from the American Type Culture Collection and were maintained in either DMEM (SKOV-3, OVCAR-3, OVCAR-8, CAOV-3 and IGROV-1), McCoy's 5a Medium Modified (HT-29 and ES-2) or RPMI-1640 (2008, RMG-1, ECC-1 and HCH-1), supplemented with 10% fetal calf serum (or 20% for OVCAR-3), penicillin (100 units/mL), and streptomycin (100 μg/mL) at 37°C with 5% CO2 in a humidified incubator. Reagents were obtained as follows: Trypsin (Sigma-Aldrich cat. #: T1426). HE4 (Fujirebio); SLIGKV-NH_2_ (Tocris cat. #: 3010); A2M (Prospec cat. #: PRO-551); TATI (R&D systems cat. #: 7496-PI); SLPI (R&D systems cat. #: 1274-PI); PAR-2 Antibody (Santa Cruz Biotechnology cat. #: sc-13504); ERK and phospho-ERK antibodies (Cell Signaling Technology cat. #: 9102 and 4370 respectively).

### Determination of cell proliferation

The indicated cells were incubated in serum-free DMEM (Gibco cat. #: 11885) overnight. The cells were treated with either trypsin or SLIGKV at the concentrations and durations indicated. Trypsin was incubated with or without HE4 in serum-free DMEM at 37°C for 24 hours. The pre-incubated culture was added to OVCAR-3 cell culture at 1:1 ratio. The culture medium and treatment were replenished once after 48 hours unless otherwise mentioned. Following the treatment, cell proliferation was measured by the MTS (Promega cat. #: G3581) assay.

### Immunoblotting and protein gel staining

Cells were treated under the conditions indicated. For the PAR2 knock-down experiments, OVCAR-3 cells were transfected with non-targeting control or PAR2 targeting siRNAs (Dharmacon: D-001210-03, D-005095-03 or D-005095-04) using Lipofectamine 3000 (Invitrogen). Following the treatment, cell lysates were collected and subjected to Western blot analysis using the protocol previously published [44]. Protein staining was performed by a silver stain kit (Pierce), following the manufacturer’s protocol.

### Reverse-transcription PCR (RT-PCR)

Total RNAs were isolated from the indicated cells using TRI Reagent and Direct-zol (Zymo Research) and were reverse transcribed using the iScript cDNA synthesis kit (BioRad), following the manufacturer’s recommendations. PCR amplification was performed as follows; 95°C for 5m, 95°C for 40s, 54° for 30s, 72°C for 30s, 72°C for 7m; 35 cycles; PRSS1 (forward: 5’-CCA CCC CCA ATA CGA CAG GAA G-3’; reverse: 5’-GCG CCA GAG CTC GCA GT-3’; 170 bp), PRSS2 (forward: 5’-CCA AAT ACA ACA GCC GG-3’; reverse: 5’-AGT CGG CAC CAG AAC TCA GA-3’; 171 bp), GAPDH (forward: 5’-AAT CCC ATC ACC ATC TTC C-3’; reverse: 5’-GTC CTT CCA CGA TAC CAA AG-3’; 307 bp). PCR-amplified samples were resolved on 2% agarose gel and visualized by SYBR Safe DNA gel stain (Invitrogen).

### Trypsin activity assay

Trypsin was co-incubated with other proteins under the conditions as follows; trypsin (150 nM) was incubated with either SLPI (300 nM; R&D systems cat. #: 1274-PI) or HE4 (100 nM) in Dulbecco's PBS (Gibco cat. #: 14040). A2M (75 nM; Prospec cat. #: PRO-551) was incubated with trypsin (75 nM) for 1 hour at 37°C in TCNB buffer (50 mM Tris, 10 mM CaCl2, 150 mM NaCl, 0.05% Brij-35 (v/v), pH 7.5). TATI (225 nM; R&D systems cat. #: 7496-PI) was incubated with trypsin (75 nM) for 30 min at room temperature in TCNB buffer. Trypsin activity was measured by the proteolytic cleavage of its chromogenic substrate *N*_α_-Benzoyl-DL-arginine *p*-nitroanilide HCl (Sigma-Aldrich cat. #: B4875; at 1 mM for SLPI and HE4 co-incubation study or at 300 μM for A2M and TATI), which releases p-nitroanilide that was monitored at 415 nm using a spectrophotometer.

### Trypsin enzyme-linked immunosorbent assay and tissue sample RT-PCR

Blood and tissue samples were obtained with written informed consent under a protocol approved by the Institutional Review Board at the University of Rochester. Serum levels of trypsin in patients with ovarian cancer were analyzed by human pan-trypsin specific enzyme-linked immunosorbent assay (R&D systems cat. #: DY3586) following the manufacturer’s protocol without further optimization. This kit recognizes human trypsin 1/2/3 isoforms (as in both pro and mature forms; see the manufacturer’s datasheet). Each sample was read in duplicate and its trypsin level was interpolated by the readings from standards. Serum HE4 was quantitatively measured by electrochemiluminescent immunoassay on a Cobas 601 system (Roche). Tissues were homogenized in buffer RLT using a mechanical grinder and QIAshredder (Qiagen). The purification of RNAs was carried out using RNeasy Plus Mini kit (Qiagen). RT-PCR was performed as described above.

### Genomics data and statistical analysis

The relative expression of PAR isoforms in ovarian cancer or tissue distribution of TF or trypsin-1/2 between OSE and ovarian cancer tissues was analyzed using the TCGA (Tumor Ovarian Serous Cystadenocarcinoma-TCGA-527-MAS5-htu133a) or Mixed Ovarian Cancer (CAFs)-Wong-77-MAS5.0-u133p2 dataset respectively. The datasets were acquired through 'R2: Genomics Analysis and Visualization Platform (http://r2.amc.nl)'. All the statistical analyses were performed using Prism 8 software (GraphPad).

## Supporting information

S1 Fig Relative gene expression of PARs in ovarian cancer was analyzed from the datasets including (A) Mixed Ovarian Cancer (CAFs)-Wong-77-MAS5.0-u133p2; N = 32; ****: p<0.0001: unpaired t test (PAR2 vs. PAR1) or Welch’s t test (PAR3 vs. PAR2; PAR4 vs. PAR2), (B) Mixed Ovarian-Birrer-63-MAS5.0-u133p2; N = 53; p<0.0001: unpaired t test (PAR2 vs. PAR1; PAR4 vs. PAR2) or Welch’s t test (PAR3 vs. PAR2) and (C) Tumor Ovarian-Bowtell-285-MAS5.0-u133p2; N = 285 p<0.0001: unpaired t test (PAR2 vs. PAR1) or Welch’s t test (PAR3 vs. PAR2; PAR4 vs. PAR2); Violin plot: median (dashed line); quartiles (dotted line).
(TIF)

S2 Fig Tissue distribution of PAR2 activating factors between OSE and ovarian cancer tissues was analyzed from the Mixed Ovarian-Birrer-63-MAS5.0-u133p2 dataset; (OSE: Ovarian Surface Epithelium, *N* = 10; ovarian tumor: papillary serous ovarian adenocarcinoma, *N* = 53); ns: not significant; ****: *p*<0.0001 (unpaired t test); TF: tissue factor. (TIF)

S3 Fig Trypsin activity was measured by the proteolytic cleavage of its substrate (*N*_α_-benzoyl-DL-arginine *p*-nitroanilide). The release of *p*-nitroanilide was colorimetrically detected at λ = 415 nm. Tr: trypsin (150 nM); HE4 (100 nM; Novoprotein Cat. #: c550).
(TIF)

S1 Raw images (DOCX)

## References

[1] Lopez-Otin C, Bond JS. Proteases: multifunctional enzymes in life and disease. J Biol Chem. 2008;283(45):30433–7. 10.1074/jbc.R800035200 18650443 PMC2576539

[2] Kim J, Yu W, Kovalski K, Ossowski L. Requirement for specific proteases in cancer cell intravasation as revealed by a novel semiquantitative PCR-based assay. Cell. 1998;94(3):353–62. 10.1016/s0092-8674(00)81478-6 9708737

[3] Turk B. Targeting proteases: successes, failures and future prospects. Nat Rev Drug Discov. 2006;5(9):785–99. 10.1038/nrd2092 16955069

[4] Mohamed MM, Sloane BF. Cysteine cathepsins: multifunctional enzymes in cancer. Nat Rev Cancer. 2006;6(10):764–75. 10.1038/nrc1949 16990854

[5] Friedl P, Wolf K. Tumour-cell invasion and migration: diversity and escape mechanisms. Nat Rev Cancer. 2003;3(5):362–74. 10.1038/nrc1075 12724734

[6] Williams SJ, Gotley DC, Antalis TM. Human trypsinogen in colorectal cancer. Int J Cancer. 2001;93(1):67–73. 10.1002/ijc.1304 11391623

[7] Miyata S, Miyagi Y, Koshikawa N, Nagashima Y, Kato Y, Yasumitsu H, et al Stimulation of cellular growth and adhesion to fibronectin and vitronectin in culture and tumorigenicity in nude mice by overexpression of trypsinogen in human gastric cancer cells. Clin Exp Metastasis. 1998;16(7):613–22. 10.1023/a:1006576313979 9932608

[8] Kawano N, Osawa H, Ito T, Nagashima Y, Hirahara F, Inayama Y, et al Expression of gelatinase A, tissue inhibitor of metalloproteinases-2, matrilysin, and trypsin(ogen) in lung neoplasms: an immunohistochemical study. Hum Pathol. 1997;28(5):613–22. 10.1016/s0046-8177(97)90085-x 9158711

[9] Hirahara F, Miyagi Y, Miyagi E, Yasumitsu H, Koshikawa N, Nagashima Y, et al Trypsinogen expression in human ovarian carcinomas. Int J Cancer. 1995;63(2):176–81. 10.1002/ijc.2910630205 7591200

[10] Wojtukiewicz MZ, Hempel D, Sierko E, Tucker SC, Honn KV. Protease-activated receptors (PARs)—biology and role in cancer invasion and metastasis. Cancer Metastasis Rev. 2015;34(4):775–96. 10.1007/s10555-015-9599-4 26573921 PMC4661218

[11] Lukkonen A, Sorsa T, Salo T, Tervahartiala T, Koivunen E, Golub L, et al Down-regulation of trypsinogen-2 expression by chemically modified tetracyclines: association with reduced cancer cell migration. Int J Cancer. 2000;86(4):577–81. 10.1002/(sici)1097-0215(20000515)86:4<577::aid-ijc21>3.0.co;2-j 10797274

[12] Koivunen E, Itkonen O, Halila H, Stenman UH. Cyst fluid of ovarian cancer patients contains high concentrations of trypsinogen-2. Cancer Res. 1990;50(8):2375–8. 2180568

[13] Hirahara F, Miyagi E, Nagashima Y, Miyagi Y, Yasumitsu H, Koshikawa N, et al Differential expression of trypsin in human ovarian carcinomas and low-malignant-potential tumors. Gynecol Oncol. 1998;68(2):162–5. 10.1006/gyno.1997.4912 9514801

[14] Paju A, Sorsa T, Tervahartiala T, Koivunen E, Haglund C, Leminen A, et al The levels of trypsinogen isoenzymes in ovarian tumour cyst fluids are associated with promatrix metalloproteinase-9 but not promatrix metalloproteinase-2 activation. Br J Cancer. 2001;84(10):1363–71. 10.1054/bjoc.2001.1806 11355948 PMC2363633

[15] Luo LY, Soosaipillai A, Grass L, Diamandis EP. Characterization of human kallikreins 6 and 10 in ascites fluid from ovarian cancer patients. Tumour Biol. 2006;27(5):227–34. 10.1159/000094693 16864975

[16] Paju A, Vartiainen J, Haglund C, Itkonen O, von Boguslawski K, Leminen A, et al Expression of trypsinogen-1, trypsinogen-2, and tumor-associated trypsin inhibitor in ovarian cancer: prognostic study on tissue and serum. Clin Cancer Res. 2004;10(14):4761–8. 10.1158/1078-0432.CCR-0204-03 15269150

[17] Koivunen E, Ristimaki A, Itkonen O, Osman S, Vuento M, Stenman UH. Tumor-associated trypsin participates in cancer cell-mediated degradation of extracellular matrix. Cancer Res. 1991;51(8):2107–12. 2009530

[18] Koivunen E, Huhtala ML, Stenman UH. Human ovarian tumor-associated trypsin. Its purification and characterization from mucinous cyst fluid and identification as an activator of pro-urokinase. J Biol Chem. 1989;264(24):14095–9. 2503510

[19] Schmalfeldt B, Prechtel D, Harting K, Spathe K, Rutke S, Konik E, et al Increased expression of matrix metalloproteinases (MMP)-2, MMP-9, and the urokinase-type plasminogen activator is associated with progression from benign to advanced ovarian cancer. Clin Cancer Res. 2001;7(8):2396–404. 11489818

[20] Kobayashi H, Ohi H, Sugimura M, Shinohara H, Fujii T, Terao T. Inhibition of in vitro ovarian cancer cell invasion by modulation of urokinase-type plasminogen activator and cathepsin B. Cancer Res. 1992;52(13):3610–4. 1617632

[21] Dery O, Corvera CU, Steinhoff M, Bunnett NW. Proteinase-activated receptors: novel mechanisms of signaling by serine proteases. Am J Physiol. 1998;274(6 Pt 1):C1429–52. 10.1152/ajpcell.1998.274.6.C1429 9696685

[22] Macfarlane SR, Seatter MJ, Kanke T, Hunter GD, Plevin R. Proteinase-activated receptors. Pharmacol Rev. 2001;53(2):245–82. 11356985

[23] Trejo J. Protease-activated receptors: new concepts in regulation of G protein-coupled receptor signaling and trafficking. J Pharmacol Exp Ther. 2003;307(2):437–42. 10.1124/jpet.103.052100 12966163

[24] Darmoul D, Gratio V, Devaud H, Laburthe M. Protease-activated receptor 2 in colon cancer: trypsin-induced MAPK phosphorylation and cell proliferation are mediated by epidermal growth factor receptor transactivation. J Biol Chem. 2004;279(20):20927–34. 10.1074/jbc.M401430200 15010475

[25] Vu TK, Hung DT, Wheaton VI, Coughlin SR. Molecular cloning of a functional thrombin receptor reveals a novel proteolytic mechanism of receptor activation. Cell. 1991;64(6):1057–68. 10.1016/0092-8674(91)90261-v 1672265

[26] Hollenberg MD, Compton SJ. International Union of Pharmacology. XXVIII. Proteinase-activated receptors. Pharmacol Rev. 2002;54(2):203–17. 10.1124/pr.54.2.203 12037136

[27] Jahan I, Fujimoto J, Alam SM, Sato E, Sakaguchi H, Tamaya T. Role of protease activated receptor-2 in tumor advancement of ovarian cancers. Ann Oncol. 2007;18(9):1506–12. 10.1093/annonc/mdm190 17761706

[28] Chanakira A, Westmark PR, Ong IM, Sheehan JP. Tissue factor-factor VIIa complex triggers protease activated receptor 2-dependent growth factor release and migration in ovarian cancer. Gynecol Oncol. 2017;145(1):167–75. 10.1016/j.ygyno.2017.01.022 28148395 PMC5359044

[29] Nemoda Z, Sahin-Toth M. Chymotrypsin C (caldecrin) stimulates autoactivation of human cationic trypsinogen. J Biol Chem. 2006;281(17):11879–86. 10.1074/jbc.M600124200 16505482 PMC1586167

[30] Szmola R, Sahin-Toth M. Chymotrypsin C (caldecrin) promotes degradation of human cationic trypsin: identity with Rinderknecht's enzyme Y. Proc Natl Acad Sci U S A. 2007;104(27):11227–32. 10.1073/pnas.0703714104 17592142 PMC2040881

[31] Moore RG, Brown AK, Miller MC, Skates S, Allard WJ, Verch T, et al The use of multiple novel tumor biomarkers for the detection of ovarian carcinoma in patients with a pelvic mass. Gynecol Oncol. 2008;108(2):402–8. 10.1016/j.ygyno.2007.10.017 18061248

[32] Kirchhoff C, Habben I, Ivell R, Krull N. A major human epididymis-specific cDNA encodes a protein with sequence homology to extracellular proteinase inhibitors. Biol Reprod. 1991;45(2):350–7. 10.1095/biolreprod45.2.350 1686187

[33] Clauss A, Lilja H, Lundwall A. A locus on human chromosome 20 contains several genes expressing protease inhibitor domains with homology to whey acidic protein. Biochem J. 2002;368(Pt 1):233–42. 10.1042/BJ20020869 12206714 PMC1222987

[34] Sallenave JM. The role of secretory leukocyte proteinase inhibitor and elafin (elastase-specific inhibitor/skin-derived antileukoprotease) as alarm antiproteinases in inflammatory lung disease. Respir Res. 2000;1(2):87–92. 10.1186/rr18 11667971 PMC59548

[35] McCubrey JA, Steelman LS, Chappell WH, Abrams SL, Wong EW, Chang F, et al Roles of the Raf/MEK/ERK pathway in cell growth, malignant transformation and drug resistance. Biochim Biophys Acta. 2007;1773(8):1263–84. 10.1016/j.bbamcr.2006.10.001 17126425 PMC2696318

[36] Nishibori M, Mori S, Takahashi HK. Physiology and pathophysiology of proteinase-activated receptors (PARs): PAR-2-mediated proliferation of colon cancer cell. J Pharmacol Sci. 2005;97(1):25–30. 10.1254/jphs.fmj04005x5 15655297

[37] Cowden Dahl KD, Symowicz J, Ning Y, Gutierrez E, Fishman DA, Adley BP, et al Matrix metalloproteinase 9 is a mediator of epidermal growth factor-dependent e-cadherin loss in ovarian carcinoma cells. Cancer Res. 2008;68(12):4606–13. 10.1158/0008-5472.CAN-07-5046 18559505 PMC3621086

[38] Hudson LG, Moss NM, Stack MS. EGF-receptor regulation of matrix metalloproteinases in epithelial ovarian carcinoma. Future Oncol. 2009;5(3):323–38. 10.2217/fon.09.10 19374540 PMC2709955

[39] Moore RG, Brown AK, Miller MC, Badgwell D, Lu Z, Allard WJ, et al Utility of a novel serum tumor biomarker HE4 in patients with endometrioid adenocarcinoma of the uterus. Gynecol Oncol. 2008;110(2):196–201. 10.1016/j.ygyno.2008.04.002 18495222 PMC3594093

[40] James NE, Chichester C, Ribeiro JR. Beyond the Biomarker: Understanding the Diverse Roles of Human Epididymis Protein 4 in the Pathogenesis of Epithelial Ovarian Cancer. Front Oncol. 2018;8:124 10.3389/fonc.2018.00124 29740539 PMC5928211

[41] Kirchhoff C. Molecular characterization of epididymal proteins. Rev Reprod. 1998;3(2):86–95. 10.1530/ror.0.0030086 9685187

[42] Bingle L, Cross SS, High AS, Wallace WA, Rassl D, Yuan G, et al WFDC2 (HE4): a potential role in the innate immunity of the oral cavity and respiratory tract and the development of adenocarcinomas of the lung. Respir Res. 2006;7:61 10.1186/1465-9921-7-61 16600032 PMC1459147

[43] Hua L, Liu Y, Zhen S, Wan D, Cao J, Gao X. Expression and biochemical characterization of recombinant human epididymis protein 4. Protein Expr Purif. 2014;102:52–62. 10.1016/j.pep.2014.08.004 25131860

[44] Kim KK, Abelman S, Yano N, Ribeiro JR, Singh RK, Tipping M, et al Tetrathiomolybdate inhibits mitochondrial complex IV and mediates degradation of hypoxia-inducible factor-1alpha in cancer cells. Sci Rep. 2015;5:14296 10.1038/srep14296 26469226 PMC4606568

