# Supplementary figures and images for: Role of trypsin and protease-activated receptor-2 in ovarian cancer
Source: PLoS One. 2020 May 4;15(5):e0232253. doi: 10.1371/journal.pone.0232253 (PMC7197761; doi:10.1371/journal.pone.0232253)

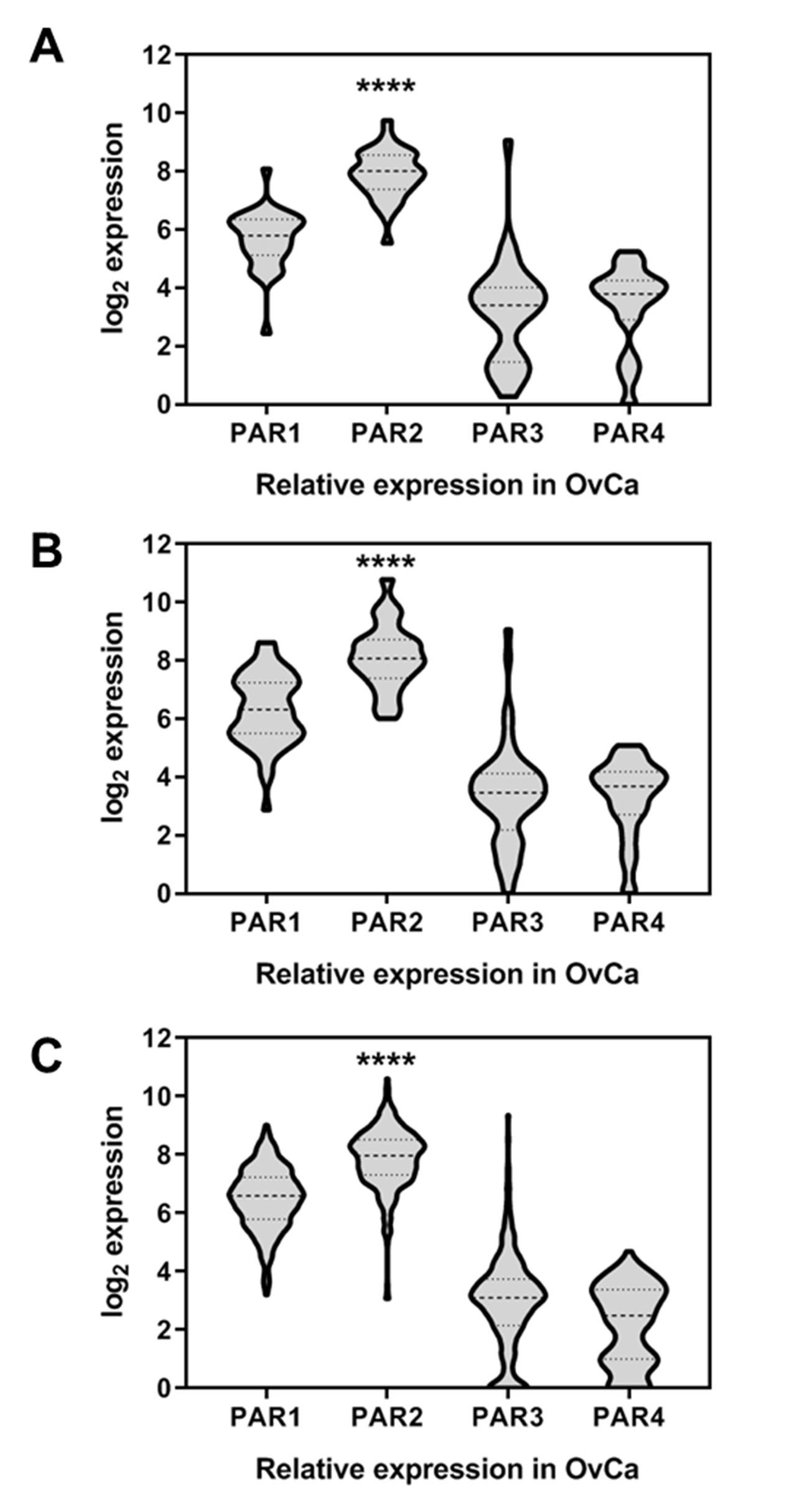

Supplement: S1 Fig — Relative gene expression of PARs in ovarian cancer was analyzed from the datasets including (A) Mixed Ovarian Cancer (CAFs)-Wong-77-MAS5.0-u133p2; N = 32; ****: p<0.0001: unpaired t test (PAR2 vs. PAR1) or Welch’s t test (PAR3 vs. PAR2; PAR4 vs. PAR2), (B) Mixed Ovarian-Birrer-63-MAS5.0-u133p2; N = 53; p<0.0001: unpaired t test (PAR2 vs. PAR1; PAR4 vs. PAR2) or Welch’s t test (PAR3 vs. PAR2) and (C) Tumor Ovarian-Bowtell-285-MAS5.0-u133p2; N = 285 p<0.0001: unpaired t test (PAR2 vs. PAR1) or Welch’s t test (PAR3 vs. PAR2; PAR4 vs. PAR2); Violin plot: median (dashed line); quartiles (dotted line). (TIF) [file pone.0232253.s001.tif]

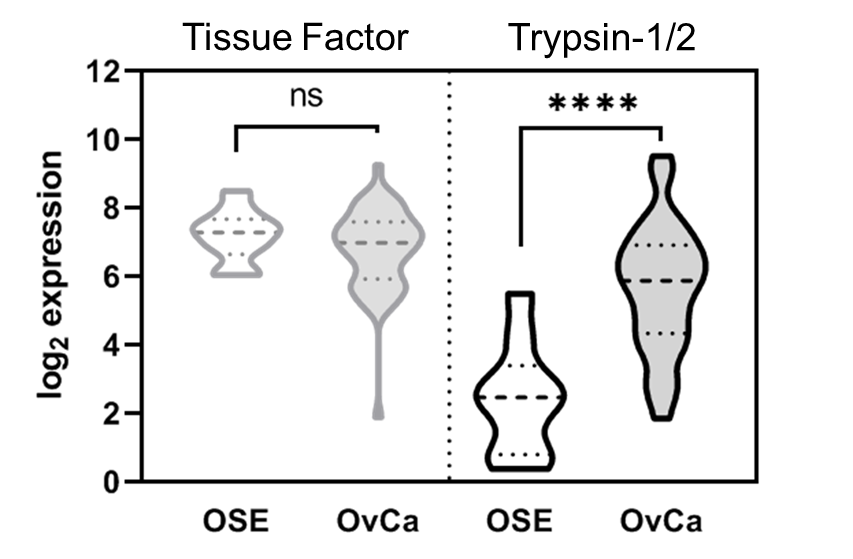

Supplement: S2 Fig — (TIF) [file pone.0232253.s002.tif]

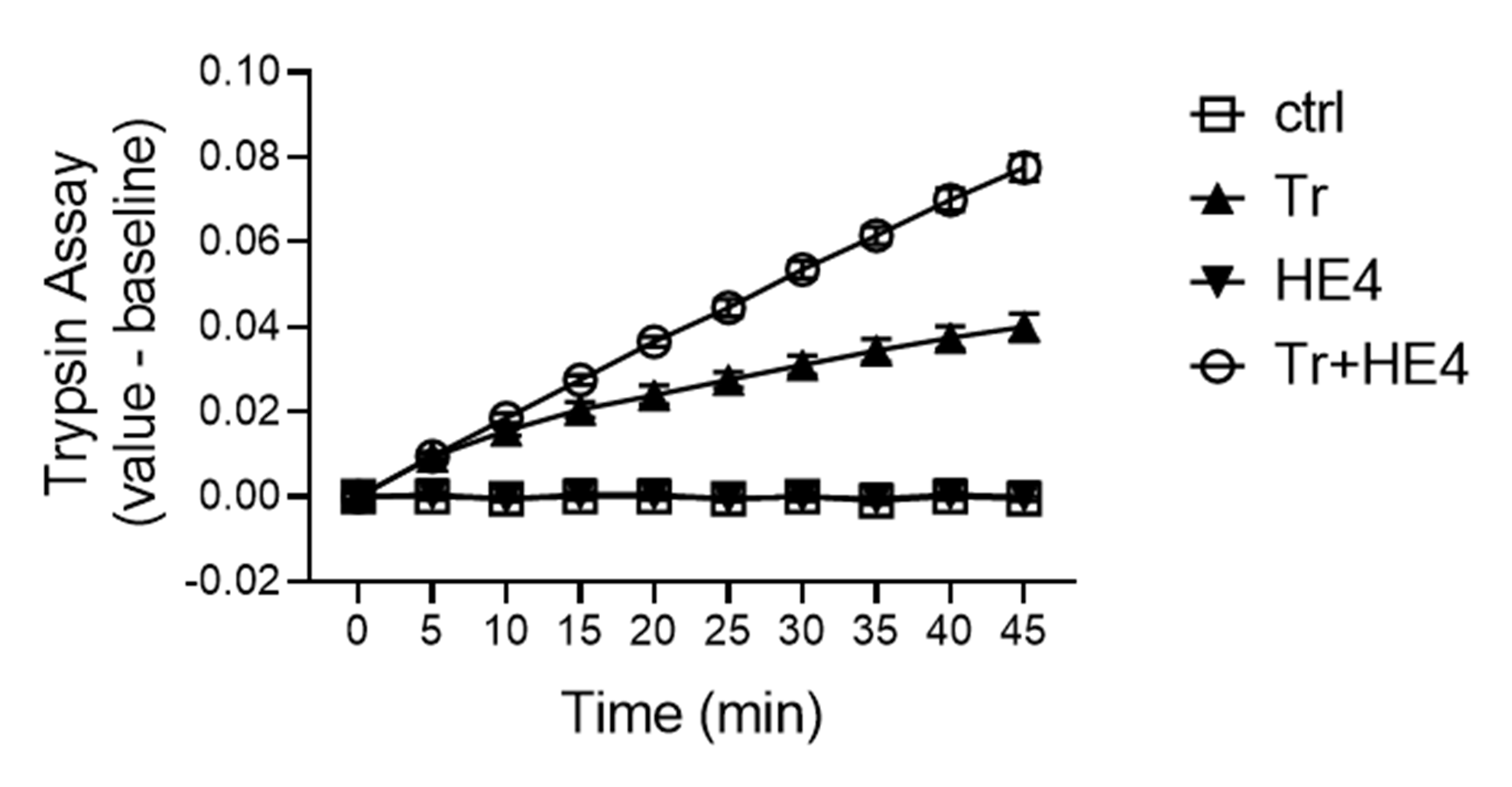

Supplement: S3 Fig — The release of p-nitroanilide was colorimetrically detected at λ = 415 nm. Tr: trypsin (150 nM); HE4 (100 nM; Novoprotein Cat. #: c550). (TIF) [file pone.0232253.s003.tif]

Fig1 C


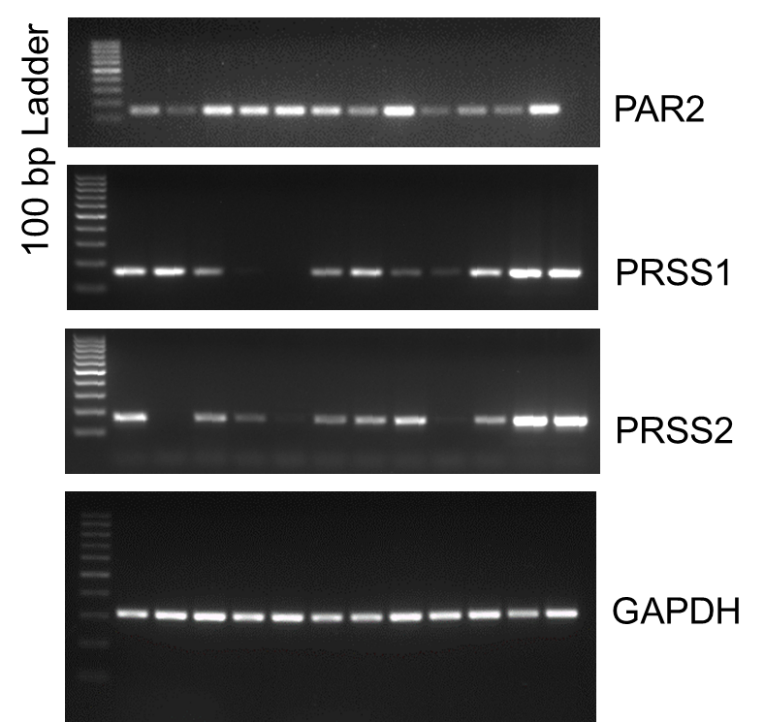


Fig2 A


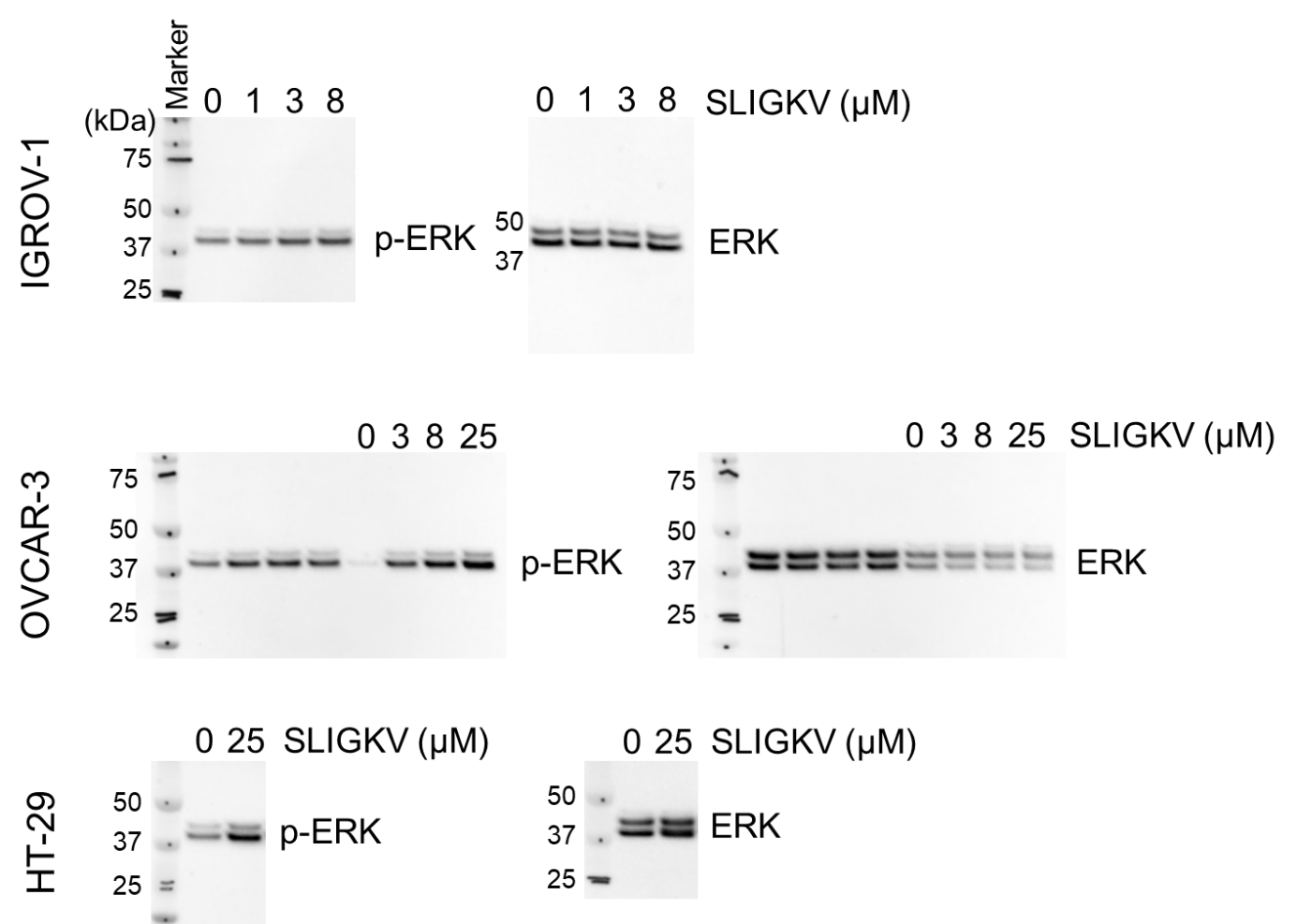


Fig2 B


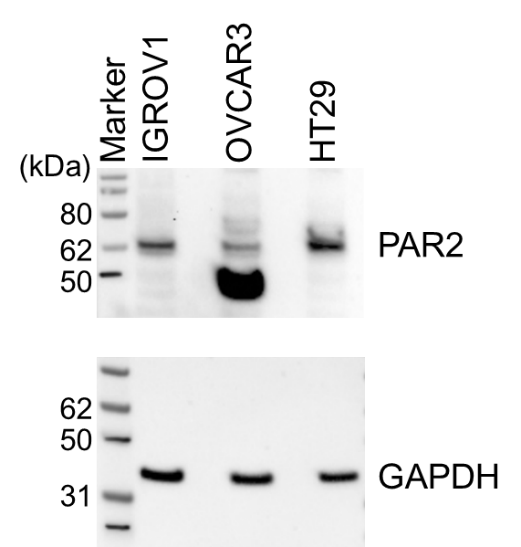


Fig2 E


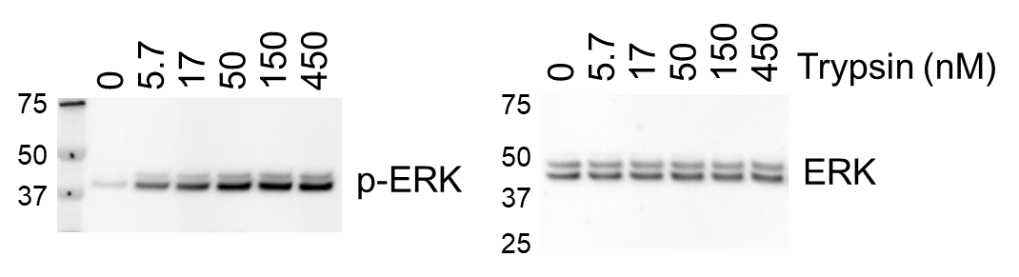


Fig2 F (Top)


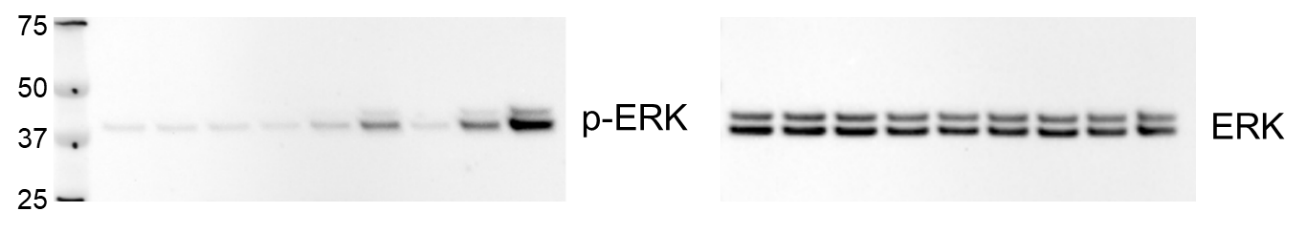


Fig2 F (Bottom)


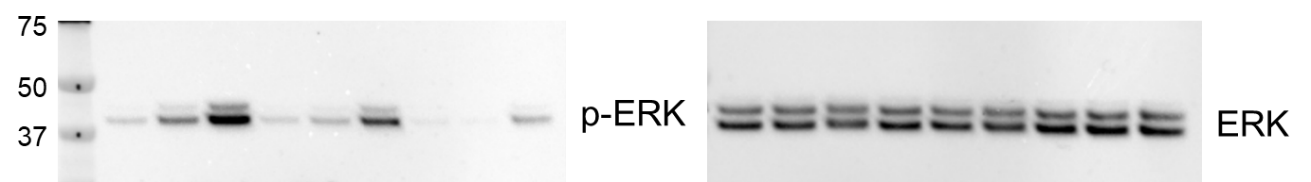


Fig2 G


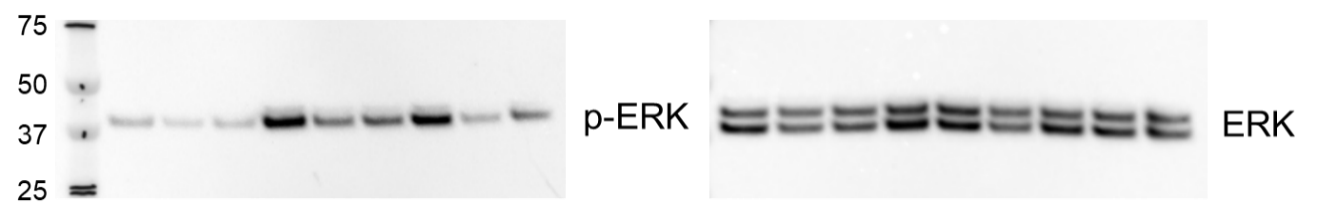


Fig5 D


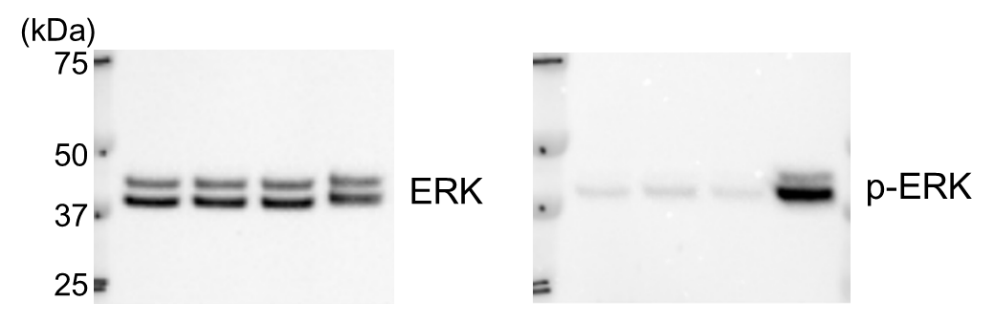


Fig6 B


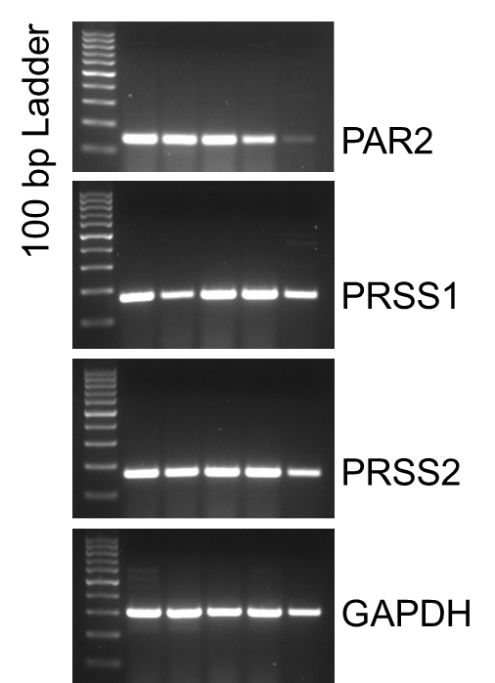

Supplement: S1 Raw images — (DOCX) [file pone.0232253.s004.docx]
